# Deeper Caribbean reef fish communities show greater taxonomic and functional change in dominance structure over a nine-year period

**DOI:** 10.1007/s00338-025-02709-7

**Published:** 2025-09-15

**Authors:** James S. Boon, Sally A. Keith, Dan A. Exton, Erika Gress, Dominic A. Andradi-Brown, Richard Field

**Affiliations:** 1 School of Geography, University of Nottingham, Nottingham, UK; 2 Lancaster Environment Centre, Lancaster University, Lancaster, UK; 3 Operation Wallacea, Wallace House, Old Bolingbroke, Spilsby, UK; 4 College of Science and Engineering, James Cook University, Townsville, Australia; 5 Ocean Conservation, World Wildlife Fund, Washington, DC USA

**Keywords:** Deep reef refuge hypothesis, Community dynamics, Temporal variability, Mesophotic

## Abstract

Fish communities at greater depths on a reef are thought to be less affected by disturbances that more strongly impact shallower areas. As a result, these deeper communities might be expected to show less change in their diversity and composition over time compared to those in shallow water. To test this hypothesis, we analysed changes in reef fish composition at 5-15 m and 25-40 m on reefs around Utila, Honduras, across two time periods: 2014-2015 and 2022-2023. We estimated taxonomic and functional α- and β- diversity using coverage-based standardisation and Hill-Chao numbers at orders q = 0 (species richness) and q = 2 (inverse Simpson index). Results showed that the α-diversity of fish communities was more consistent at 25-40 m than at shallower depths between the two time periods. However, β-diversity of dominant species and traits (q = 2) increased at greater depths, indicating that deeper fish communities became more distinct from one another in both structure and function, as well as more different from shallower communities at the same sites. Changes in diversity also varied between sites, highlighting the role of sitespecific conditions in shaping and maintaining fish communities across depths. Overall, the findings are not consistent with the expectation that greater depth reduces temporal community variability, and they raise questions about whether depth alone can serve as a refuge for reef fish.

**Supplementary Information:**

The online version contains supplementary material available at 10.1007/s00338-025-02709-7.

## Introduction

Tropical coral reefs are hyperdiverse ecosystems that provide important ecosystem services including food, income, and coastal protection (Moberg and Folke 1999; Woodhead et al. 2019). However, they have become increasingly impacted by local and global stressors, such as overexploitation, habitat destruction, and climate change (Ban et al. 2014; Hughes et al. 2018; Mora et al. 2011). Most research has assessed the impacts of these stressors on shallow coral reefs (< 30 m depth), leading to growing interest in the potential of deeper zones (> 30 m) to offer protection to reef communities (Bongaerts et al. 2010; Glynn 1996; Lesser et al. 2009; Riegl & Piller 2003). Yet, accessing greater depths is logistically challenging, resulting in a lack of temporal data across the depth gradient, particularly on fish communities. This gap in knowledge limits our understanding of how fish communities change over time at different depths, hindering our ability to determine whether deeper reefs can effectively serve as refuges against anthropogenic threats.

Mesophotic coral ecosystems (MCEs) are reef communities found at depths of approximately 30 to 150 m, where light starts to diminish (Baldwin et al. 2018; Eyal et al. 2021; Hinderstein et al. 2010; Lesser et al. 2009). As depth increases, benthic communities change from being dominated by zooxanthellate corals and macroalgae, to sponges and heterotrophic soft corals (Lesser et al. 2019, 2018). These communities are primarily structured by the reduced solar irradiance for photosynthesis associated with increased depth, along with shifts in temperature, wave energy and sedimentation (Diaz et al. 2023b; Laverick et al. 2020; Slattery et al. 2024). As a result of these abiotic and benthic community changes, fish communities also transition from a dominance of herbivorous fish in shallow waters to a greater abundance of large-bodied carnivores in the mesophotic zone (Andradi-Brown et al. 2021, 2016; Loiseau et al. 2023; Pinheiro et al. 2016; Semmler et al. 2017). Generally, fish species richness reduces across shallow and mesophotic depths, whereas community dissimilarity and species turnover increase along the depth gradient (Andradi-Brown et al. 2016; Rocha et al. 2018). There is also strong evidence of depth specificity, with many coral reef fish species found exclusively in the lower depths of MCEs (Pinheiro et al. 2016; Rocha et al. 2018; Thresher & Colin 1986). As a result, MCEs are often separated into two zones, the upper-MCE (30-60 m) and lower-MCE (60-150 m), which broadly indicate shifts in community composition (Lesser et al. 2019; Semmler et al. 2017).

The intensity of some disturbances have been reported to decrease with increased depth, leading to the idea that deeper reef communities might serve as refuge for shallow-water fish and invertebrate communities (Assis et al. 2016; Glynn 1996; Hughes & Tanner 2000; Lesser et al. 2009). The ‘deep reef refuge’ hypothesis proposes that: (1) disturbances affecting shallow-water reefs are less severe on deeper reefs, and (2) deeper reefs can provide recruits for shallower areas, aiding recovery post-disturbance (Bongaerts et al. 2010). Research on brooding coral species suggests that lower-MCEs may have limited potential as refuge for shallow-water communities due to greater community dissimilarity and reduced population connectivity between depth zones, while upper-MCEs may have greater promise (Bongaerts et al. 2017; Brazeau et al. 2013; Slattery et al. 2024). Bongaerts and Smith (2019) described this as the ‘disturbance/divergence trade-off’, meaning even if lower-MCEs offer greater protection from disturbances, they may have limited ability to supply recruits to shallow reefs, which suggests that upper-MCEs may be the most viable refuge. Furthermore, there are growing reports of stressors like marine heatwaves, tropical storms, destructive fishing, and invasive species affecting mesophotic reefs across the entire depth range, which may cast doubt on whether these deeper habitats can truly serve as a refuge for fish communities (Andradi-Brown et al., 2017a; Diaz et al. 2023a; McWhorter et al. 2024; Rocha et al. 2018; Soares et al. 2019; Venegas et al. 2019).

Studies examining the deep reef refuge hypothesis in relation to reef fish communities over time are relatively rare. Logistical challenges make repeated surveys at depth difficult, while there is also a pressing need to document the location of unrecorded MCE habitats and undescribed species to aid conservation efforts (Turner et al. 2019). The available evidence suggests that the richness and abundance of deeper fish and benthic communities are generally less stable over time than would be expected if the deep reef refuge hypothesis were accurate (Slattery et al. 2018). This instability is particularly evident when communities are threatened by invasive species or storm damage (Abesamis et al. 2018; Lesser & Slattery 2011). However, these studies typically examine fish communities across depth using only a single diversity or dissimilarity metric, each with a fixed sensitivity to rare versus abundant species, and none have considered this in relation to functional diversity (Loiseau et al. 2023). Single metrics offer a clear but potentially restricted view, so incorporating functional diversity and different diversity orders may provide a deeper understanding of community dynamics (Loiseau et al. 2023). For instance, at mesophotic depths, fish communities may experience changes in species composition over time, but these species might share the same functional traits, offering a functional refuge for key ecosystem processes (Loiseau et al. 2023).

To address these knowledge gaps, we investigated changes in the taxonomic and functional diversity and composition of fish communities across shallow and upper-mesophotic reefs in Utila, Honduras, at two time periods spanning nine years. Like many areas of the Caribbean over recent decades, Utila’s reefs have been impacted by invasive species, ocean warming, storms and pollution (Bove et al. 2022; Gardner et al. 2003; Mumby 2009). Drawing on the deep reef refuge hypothesis, we predict that fish communities at greater depths will experience smaller temporal changes, as these areas are less affected by disturbances. Therefore, we expect to see larger shifts in both taxonomic and functional diversity at shallower depths compared to deeper ones. We also anticipate that deeper communities will show less temporal change in their taxonomic and functional composition.

## Methods

### Study location

**Fig. 1:**
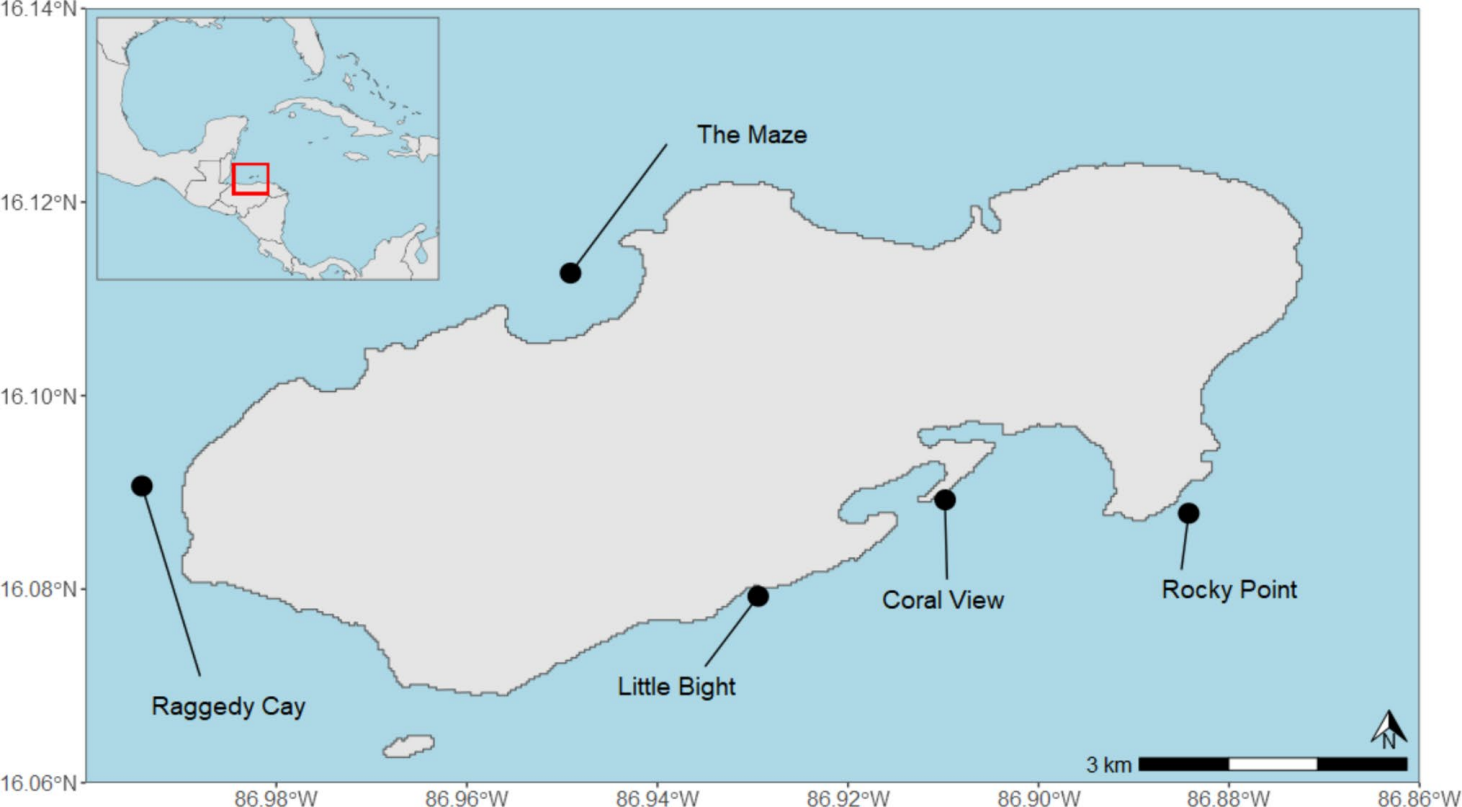
Map of study sites around the Island of Utila, Honduras. Inset map shows the location of Utila relative to the Caribbean region

Fish community data were collected at five fringing reef sites surrounding the island of Utila, Honduras (Fig. 1). Located on the southern Mesoamerican Barrier Reef, Utila is one of the three main islands and 53 cays that make up the Bay Islands Marine National Park. Two study sites, The Maze and Raggedy Cay, were situated on the island’s exposed northern shore. Reefs on the northern shore are located along a steep reef wall that quickly reaches beyond 100 m depth. The Maze and Raggedy Cay have more extensive shallow reef communities, with large back reefs behind the reef crest and greater wave exposure throughout much of the year compared to the south shore sites (Andradi-Brown et al. 2016). The other three sites - Little Bight, Coral View, and Rocky Point - were located on the more sheltered southern shore of the island. Little Bight and Coral View are the shallowest sites, where the reef slope descends gradually to 40 m before levelling out into a patch reef system in the upper-MCE. Both sites have less hard substratum available at 25 and 40 m compared with the other locations. Rocky Point on the other hand bottoms out slightly deeper, at 60 m. The reef slope angle on the southern shore sites was similar at 5, 15 and 25 m depth bands, but the seabed was flatter at 40 m depth (Andradi-Brown et al. 2016). Sites were selected based on the locations of initial surveys conducted in 2014 and 2015 to ensure continuity in data collection and allow for direct temporal comparisons. Research permits for the study were issued by the Instituto de Conservación Forestal (ICF), Honduras (Permit number: DE-MP-108-2023).

### Fish community surveys

Fish community surveys were conducted at each site at depths of 5, 15, 25, and 40 m in the years 2014, 2015, 2022, and 2023. All surveys were conducted between June and August. Surveys consisted of a calibrated diver-operated stereo-video system (SVS; SeaGIS, Melbourne, Australia) along a 50 m transect that followed the reef contour (Goetze et al. 2019). Transects were spaced with 10 m intervals between replicates. Each transect was surveyed by a team comprising an SVS operator and a second diver responsible for measuring the distance. The cameras, angled approximately 20 degrees downward, were maintained around 0.5 m above the seabed or away from the reef wall during filming. Each 50 m transect took approximately 5 min to complete. In 2014 and 2015, the SVS setup included two Canon HFS21 high-definition video cameras. This equipment was changed to two GoPro Hero 8 cameras for the 2022 and 2023 surveys. Divers conducting surveys at 40 m used mixed-gas open-circuit SCUBA, while shallower surveys used regular air. In all years, the SVS and camera systems were calibrated using a calibration cube and CAL software (http://www.seagis.com.au/bundle.html). Due to logistical constraints, no more than three transects could be conducted at a given depth per dive, as time and air limitations restricted survey time. Therefore, transects at each depth were spread across multiple dives, minimising pseudoreplication. To avoid resampling and ensure each transect was distinct, divers consistently swam either to the left or right of the site buoy along the reef slope, starting where any previous transects had ended.

Footage was analysed using EventMeasure software (SeaGIS, Melbourne, Australia), which allowed for the synchronisation of the calibrated SVS footage and measurement of fish total lengths. As the video moved through the transect, fish located outside of 2.5 m to either side or 5 m in front of the cameras were excluded from analysis as they were not within the transect perimeter. All fish visible within these transects were recorded to species-level where possible, using Humann and Deloach (2014), and had their lengths measured. In cases where measurements could not be taken (e.g., when the fish was not in a clear line of site of the cameras), the species was recorded, and the average length for that species at the same site, depth, and time was used. If no other individuals of that species were observed under those conditions, the average length for that species across all sites and depths for that year was used.

The number of 50 m transects conducted at each site, depth, and time point ranged from 1 to 12, with a median of 8 (Tables S1 and S2). Fewer transects were completed at 25 and 40 m due to the increased logistical challenges of deeper diving. In 2022-2023, these constraints limited sampling at 40 m to a single transect each at Little Bight and Coral View. To ensure a sufficient number of replicates for subsequent analysis, species abundance data from all transects at each site were pooled into two time periods (2014 to 2015 and 2022 to 2023) and two depth categories (5-15 and 25-40 m). While binning depth, a continuous variable, may obscure finer-scale patterns, this approach was necessary to ensure sufficient initial sample coverage for more reliable estimates of community diversity.

### Taxonomic and functional α-diversity

To estimate and compare taxonomic and functional diversity, sampling units were standardised to account for the uneven number of transects across sites, depths, and time periods (Chao and Jost 2012). To achieve this, a sample coverage approach was used, which standardises diversity estimates based on the proportion of observed diversity relative to the estimated true diversity (Chao et al. 2021). Diversity estimates were standardised following the non-asymptotic approach recommended by Chao and Jost (2012), using the *iNEXT.3D* (Chao et al. 2021) package in R v. 4.2.3 (R Core Team, 2023). These estimates were calculated using Hill-Chao numbers, which represent the effective number of equally abundant species, for orders q = 0 and q = 2. Order q = 0 corresponds to species or functional richness, reflecting the number of species or traits present in the community, but does not account for their abundances, meaning it gives more weight to rarer species or traits (Chao et al. 2021). Order q = 2 corresponds to the inverse of the Simpson index, which emphasises the most dominant species or traits, meaning it gives more weight to highly abundant species or traits in the community (Chao et al. 2021). These two Hill-Chao numbers represent the two extremes of the diversity spectrum and clearly highlight different patterns in community composition.

Taxonomic and functional α-diversity were estimated for fish communities at each of the five sites, depths (5-15 and 25-40 m), and time (2014/15 and 2022/23) combination. To reduce prediction bias, all estimates for q = 0 were calculated to the coverage of double the reference sample size. This is equal to the sample coverage of the time, site and depth group with the lowest coverage, which is then extrapolated to twice the original number (Chao et al. 2021). In our case, standardised coverage C_max_ for q = 0 was 97.16%. As extrapolation for q = 2 results in only small error, diversity estimates were extrapolated to C_max_ 100% (Chao et al. 2014). In *iNEXT.3D*, the estimate3D function was used to determine taxonomic α-diversity (q = 0 and q = 2). To estimate functional α-diversity, five traits were compiled for all fish species in the dataset (Table 1). This included diet, position in the water column, body size, gregariousness and vertical home range. Information for each trait was collected to species level. Although juveniles were identified for some species, this was not possible for all due to difficulties in distinguishing juvenile stages. Therefore, intraspecific variation was not considered, and traits reflect adult characteristics. Based on these functional traits, the multidimensional trait distance (Gower distance) between species was calculated (Gower 1971) using the mFD package in R (Magneville et al. 2022). To estimate functional α-diversity at each site, depth and time combination, the estimate3D function was used with the abundance dataset and the Gower functional distance matrix, using the default Fdtype = ‘AUC’ (area under the curve of the tau profile).

**Table 1:** Description of traits used to determine functional diversity and dissimilarity

Trait name	Ecological relevance	Trait levels	References
Diet	The diet of a fish shapes which ecosystem processes they influence (Bellwood et al. 2005; Sale 1977). For example, herbivores feed on algae, which helps prevent fleshy macroalgae from dominating the reef, allowing reef-building benthic organisms to persist, and reducing the risk of regime shifts	Herbivore, Planktivore, Carnivore, Invertivore, Omnivore, Piscivore	Froese and Pauly (2024); Humann and Deloach (2014)
Body size	Body size is used to define a species niche as it can influence energy requirements, growth rates, and predator–prey interactions due to mouth gape and body size relationship (Fisher et al. 2010)	Average body size for each species. Continuous (cm)	Estimate calculated from our dataset
Position in the water column	A measure of fish association with the reef matrix and the set of potential prey available (Bellwood et al. 2005)	Benthic, Benthopelagic, Pelagic	Froese and Pauly (2024); Humann and Deloach (2014)
Vertical home range (depth range where the species is known to occur)	Reflects the ability to move across different depth zones, influencing ecological connectivity and resource use within reef ecosystems (Slattery et al. 2011; Stefanoudis et al. 2019)	Continuous (m)	Froese and Pauly (2024)
Gregariousness	Behavioural trait that influence fish anti-predator responses (Stier et al. 2013). Schooling species can impact nutrient cycling and local resource depletion (Foster 1985)	Solitary, pairing, small group of 3–20 individuals, medium group of 20–50 individuals, and large group > 50 individuals	Quimbayo et al. (2021)

For both taxonomic and functional α-diversity, diversity estimates were plotted with 95% confidence intervals (CIs). Statistical significance at 5% can conservatively be inferred where CIs do not overlap (Chao et al. 2014), as per Cumming et al. (2007), Diaz et al (2024), Gorta et al. (2023) and Hacala et al. (2024). The outputs were plotted in a point plot with ggplot2 package in R (R Core Team, 2023).

### Taxonomic and functional β-diversity

Β-diversity estimates were standardised by sample coverage using the *iNEXT.beta3D* package (Chao et al. 2023). This applies the multiplicative decomposition method to calculate β-diversity, defined as the ratio of γ-diversity to α-diversity (Chao et al. 2023; Whittaker 1972, 1960), using our abundance dataset. Taxonomic and functional β-diversity of fish communities were assessed based on Hill numbers with order q = 0 and q = 2 (Chao et al. 2019). The same traits and Gower functional distance matrix used to explore functional α-diversity were used to determine functional β-diversity (Table 1). When using *iNEXT.beta3D,* β-diversity varies between 1 and 2, with values close to 2 revealing a high difference in the taxonomic or functional composition of fish communities, while a low dissimilarity (close to 1) denotes more similar fish communities.

Taxonomic and functional β-diversity (q = 0 and q = 2) were calculated for communities from the same site and depth group across the two time periods. Then, comparisons were made between depth groups and time periods within the same site. All estimates were standardised to C_max_ 97.16% for q = 0 and C_max_ 100% for q = 2 (Chao et al. 2023). The output from *iNEXT.beta3D* provided taxonomic and functional β-diversity estimates with 95% CIs. Significant differences were inferred where CIs did not overlap, following a conservative approach to account for multiple comparisons (Chao et al. 2023). The outputs were plotted in a point plot and heatmap with ggplot2 package in R (R Core Team, 2023).

## Results

### Taxonomic and functional α-diversity

Across the five sites around Utila, a total of 33,604 reef fish were recorded during the two survey periods: 16,721 individuals in 2014/15 and 16,883 in 2022/23, at depths between 5 and 40 m. These individuals represented 29 families, 54 genera, and 119 species. In 2014/15, invasive lionfish (*Pterois volitans*) were observed nine times across four sites (Coral View, Little Bight, Raggedy Cay, and Rocky Point), all at depths of 25-40 m. In contrast, no lionfish were recorded at any site or depth in 2022/23.

**Fig. 2:**
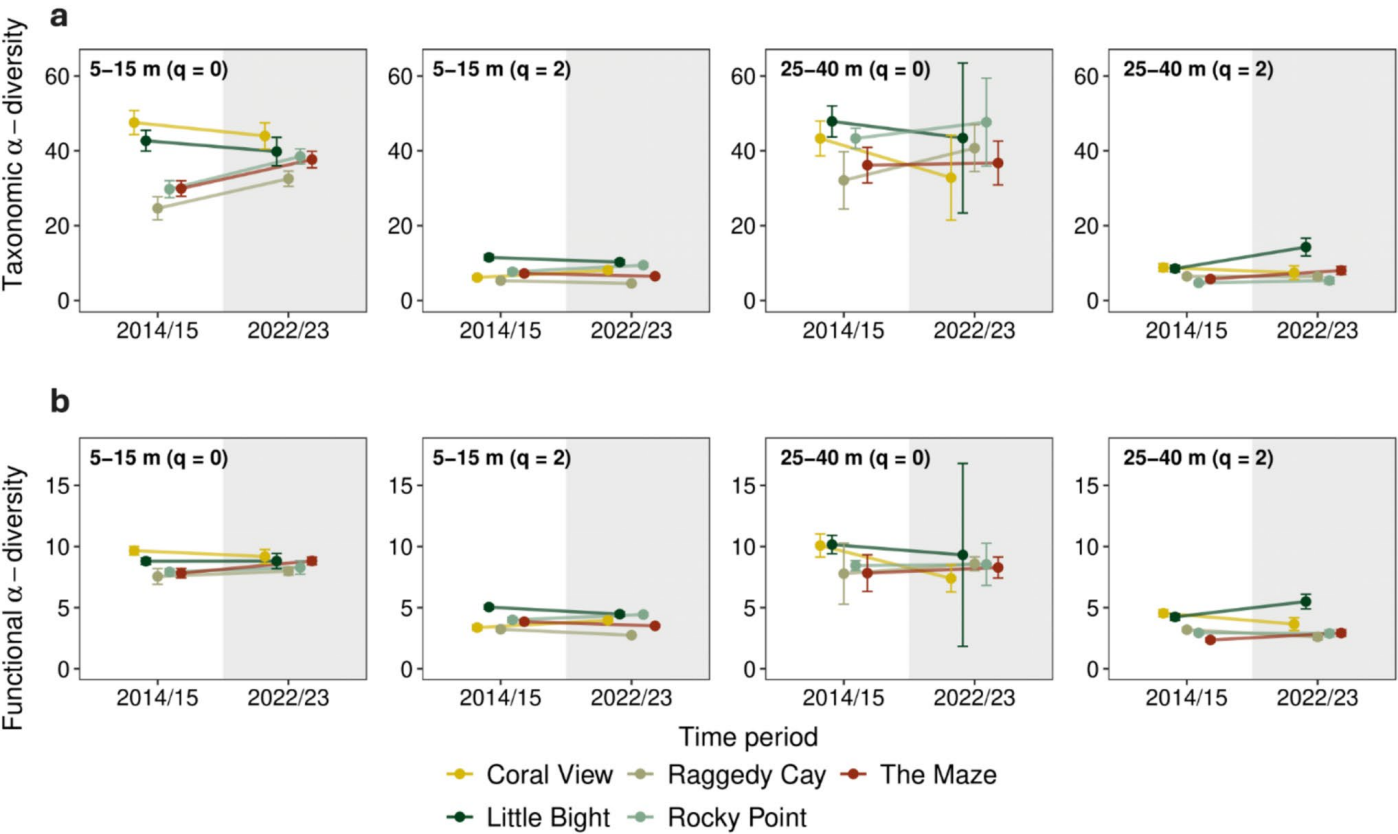
Shifts in **a** taxonomic and **b** functional α-diversity (Hill numbers q = 0 and q = 2) of fish communities at the five study sites between 2014/15 and 2022/23. Diversity estimates are shown separately for communities at 5-15 and 25-40 m depths. Error bars represent 95% CIs

Shifts in α-diversity taxonomic diversity between 2014/15 and 2022/23 were generally more prevalent at shallower depths, with variation observed among sites (Fig. 2a**; **Table S3). Species richness (q = 0) significantly increased at 5-15 m between the two time periods at three sites: Rocky Point, The Maze, and Raggedy Cay (Fig. 2a; non-overlapping 95% confidence intervals (CIs)). Whereas at 25-40 m, species richness did not change significantly between time periods at any site (overlapping 95% CIs). Taxonomic diversity (q = 2) also generally changed more at shallower depths, but with more modest shifts (Fig. 2a). At 5-15 m, taxonomic diversity (q = 2) significantly decreased at two sites (Coral View and Rocky Point) and significantly increased at two others (Raggedy Cay and The Maze; Fig. 2a; non-overlapping 95% CIs). While at 25-40 m, taxonomic diversity (q = 2) increased at two sites (Little Bight and The Maze), while the remaining sites showed no significant temporal change (overlapping 95% CIs).

Functional α-diversity (q = 0) of fish communities remained largely stable between 2014/15 and 2022/23 across sites and depths (Fig. 2b; Table S4). A significant increase was observed at The Maze at 5-15 m, and a significant decrease at Coral View at 25-40 m (non-overlapping 95% CIs), while no significant changes were detected at the other sites or depths. In contrast, functional α-diversity (q = 2) showed greater variation. At 5-15 m, values significantly decreased at three sites (The Maze, Raggedy Cay, and Little Bight) and increased at the remaining two (non-overlapping 95% CIs). At 25-40 m, functional α-diversity (q = 2) significantly decreased at Coral View and Raggedy Cay, increased at Little Bight and The Maze (non-overlapping 95% CIs), and showed no significant change at Rocky Point.

### Taxonomic and functional β-diversity

**Fig. 3:**
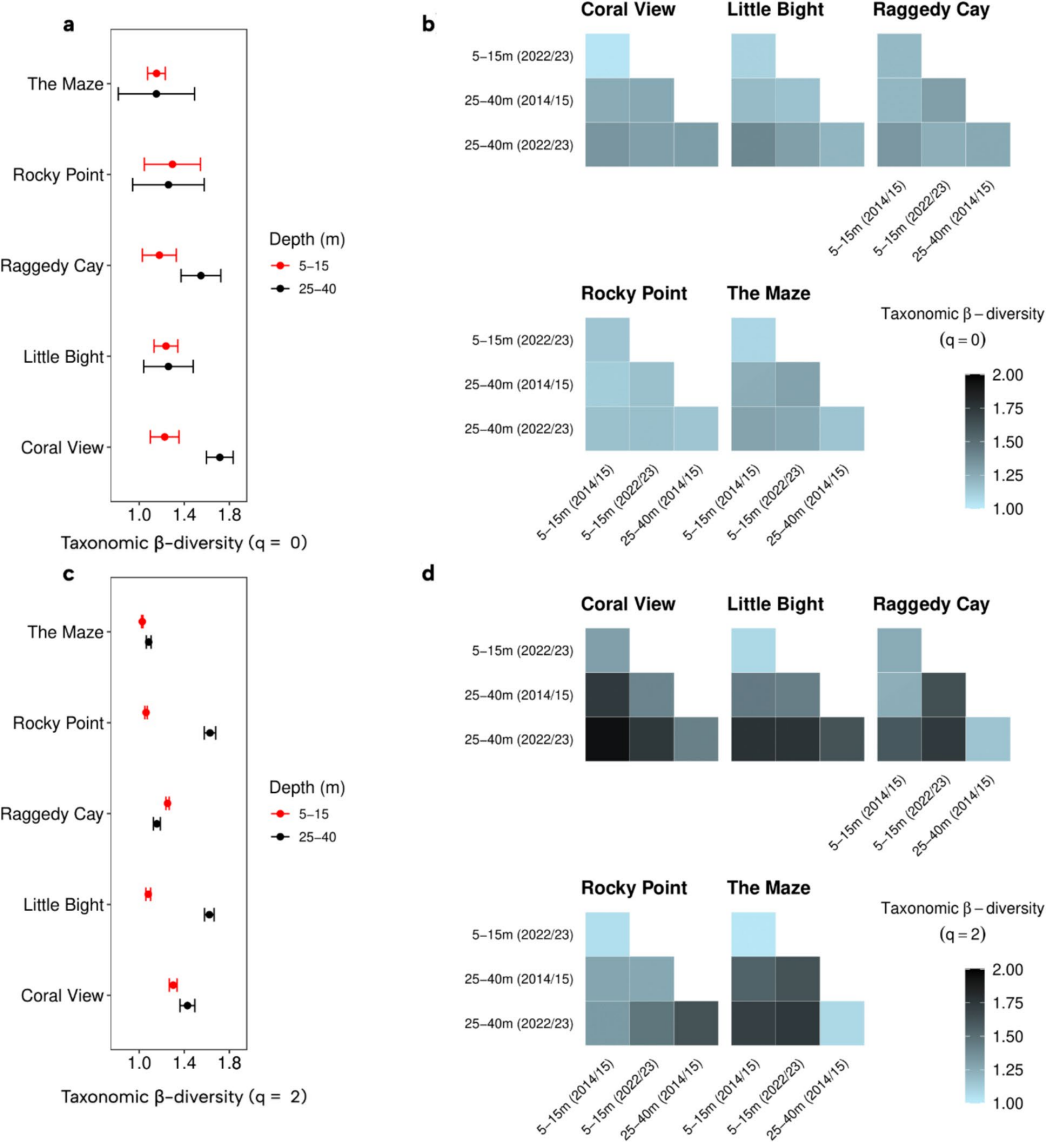
Taxonomic similarities of reef fish communities at the five study sites. Taxonomic β-diversity of fish communities based on Hill numbers. **a** q = 0 comparisons at the same site and depth between 2014/15 and 2022/23 with 95% confidence intervals. **b** q = 0 pairwise comparisons across depths and time periods within each site. **c** q = 2 comparisons at the same site and depth between 2014/15 and 2022/23 with 95% confidence intervals. **d** q = 2 pairwise comparisons across depths and time periods within each site. Values near 1 indicate high taxonomic similarity, while values near 2 indicate low similarity between communities

Taxonomic β-diversity of fish communities varied across sites, depths, and time periods, with higher values when more weight was given to abundant species (q = 2) compared to species richness alone (q = 0; Fig. 3a-d; Table S5). At Coral View and Raggedy Cay, β-diversity based on species richness (q = 0) between 2014/15 and 2022/23 was significantly higher at 25-40 m than at 5-15 m, indicating reduced community similarity at deeper depths between 2014/15 and 2022/23 (Fig. 3a). At the other three sites, there were no significant differences in similarity between depth zones. In contrast, the other four sites exhibited slightly higher β-diversity values for all depths and time combinations, though still moderate (q = 0 < 1.4; Fig. 3b). When more weight was placed on highly abundant species (q = 2), taxonomic β-diversity between 2014/15 and 2022/23 was significantly greater at 25-40 m than at 5-15 m at most sites (The Maze, Rocky Point, Little Bight and Coral View; Fig. 3c). Yet at Raggedy Cay, significantly greater values were seen at the shallower depth. At most sites, fish communities at 25-40 m in 2022/23 became less similar to shallower communities from either time period, reflected by higher β-diversity values (q = 2) at Coral View, Little Bight, Raggedy Cay, and The Maze (Fig. 3d). In contrast, at Rocky Point, fish communities at 25-40 m were more similar to shallow communities from both time periods than to each other across years.

**Fig. 4:**
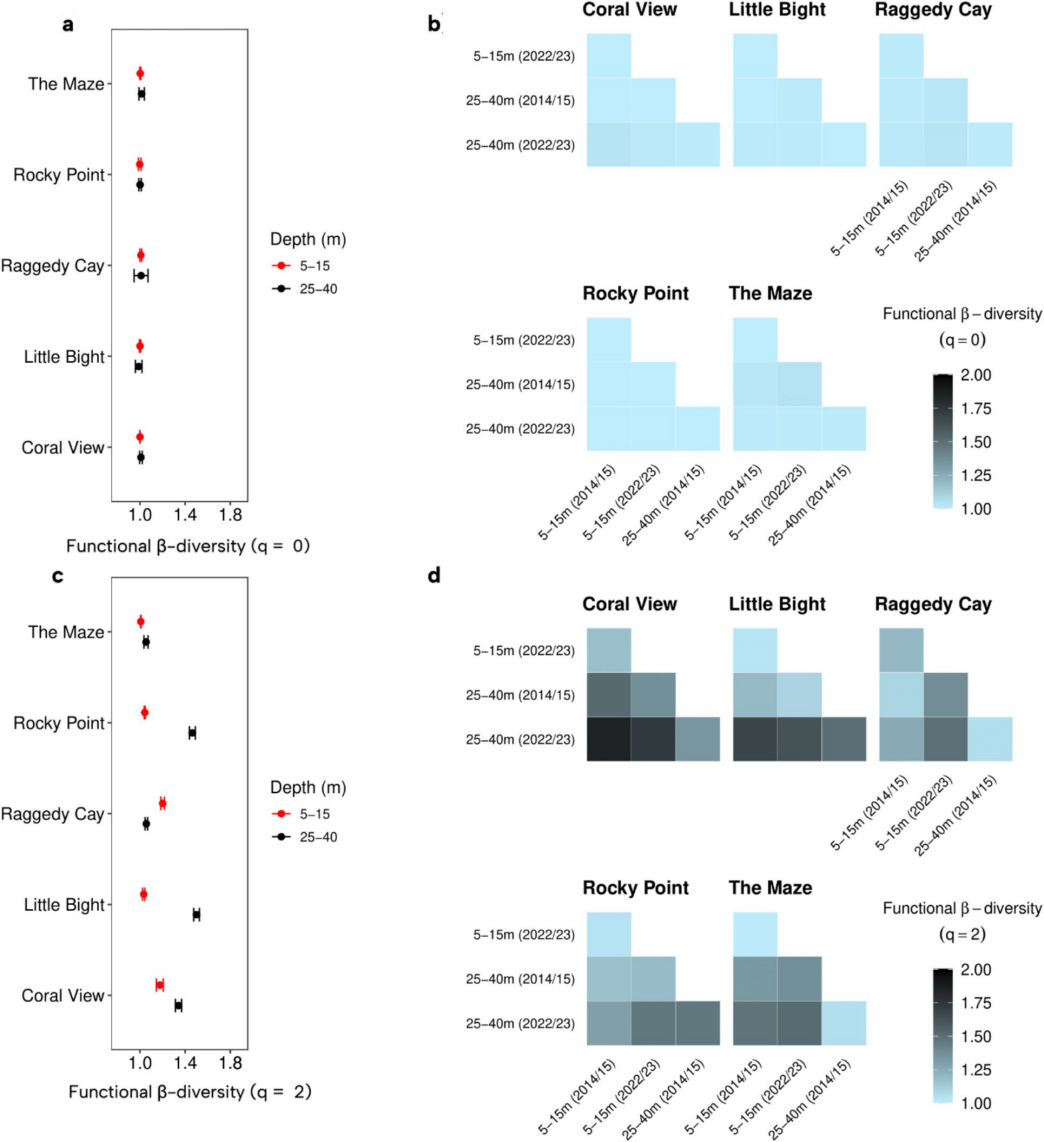
Functional similarities of reef fish communities at the five study sites. Functional β-diversity of fish communities based on Hill numbers **a** q = 0 comparisons at the same site and depth between 2014/15 and 2022/23 with 95% confidence intervals. **b** q = 0 pairwise comparisons across depths and time periods within each site. **c** q = 2 comparisons at the same site and depth between 2014/15 and 2022/23 with 95% confidence intervals. **d** q = 2 pairwise comparisons across depths and time periods within each site. Values near 1 indicate high functional similarity, while values near 2 indicate low similarity between communities

Fish community β-diversity based on functional richness (q = 0) exhibited little change across depths and sites between 2014/15 and 2022/23 (Fig. 4a, b; Table S6). Yet, when more weight was placed on highly abundant functional traits (q = 2), β-diversity exhibited greater levels of variation (Fig. 4c, d). At four out of five sites, functional β-diversity (q = 2) between 2014/15 and 2022/23 was significantly higher at 25-40 m than at 5-15 m, indicating a decline in functional similarity at deeper depths between the two time periods (Fig. 4c). Raggedy Cay was the exception, where significantly greater functional change (q = 2) was observed at the shallower depth. Within sites, functional β-diversity (q = 2) was highest between fish communities at 25-40 m in 2022/23 and those at shallower depths across both time periods at Coral View, Little Bight, and The Maze (Fig. 4d). At Raggedy Cay, fish communities at 25-40 m in 2022/23 were less functionally similar to 5-15 m communities from the same time period than to either depth from 2014/15.

## Discussion

If deeper fish communities were better protected from disturbances affecting shallow waters, they would be expected to show less temporal variation in diversity and composition. However, analysis of taxonomic and functional diversity showed that taxonomic α-diversity (q = 0 and q = 2) was generally more variable at 5-15 m, while both taxonomic and functional β-diversity of dominant species (q = 2) varied more at 25-40 m between the two time periods. This suggests that deeper fish communities became increasingly different in both the structure and function of dominant species. In addition, changes in diversity measures varied between sites, highlighting the importance of site-specific conditions in shaping and maintaining reef fish communities across depths. Overall, these results indicate that deeper fish communities around Utila have become more distinct in their dominance structure than shallow-water communities, raising questions about the assumption that deeper reefs act as a stable refuge.

Shifts in taxonomic α-diversity (q = 0 and q = 2) were greater at 5-15 m than at 25-40 m. Although the direction of these changes varied between sites, they suggest that shallow reef fish communities experienced more pronounced shifts in both species presence and relative abundance than deeper communities, which appears consistent with the deep reef refuge hypothesis (Bongaerts et al. 2010). Species richness (q = 0) increased at shallow depths at Rocky Point, The Maze, and Raggedy Cay, while no change in richness was seen at 25-40 m. This could be because new species have inhabited shallower parts of the reef from greater depths. These three sites have more continuous habitat spanning shallow and mesophotic zones, as well as greater maximum depths, compared to Coral View and Little Bight, which may facilitate vertical movement (Hollarsmith et al. 2020). This vertical connectivity supports species movement, providing access to shelter and food across depths, thereby promoting more stable and similar communities throughout the depth gradient (Abesamis et al. 2018; Slattery et al. 2011). In contrast, sites like Coral View and Little Bight, where vertical habitat is more fragmented and depth is limited, may offer less connectivity, potentially restricting species movement and limiting diversity (Serrano et al. 2022). The shifts in taxonomic diversity (q = 2) observed at shallower depths may reflect changes in community structure, with new species influencing patterns of relative abundance. However, functional richness (q = 0) remained stable across all sites and depths between 2014/15 and 2022/23, suggesting these changes in species composition do not appear to have altered the functional roles present in the ecosystem.

Although α-diversity remained relatively stable at 25-40 m, increases in β-diversity for dominant species and traits (q = 2) between the two time periods suggest that deeper fish communities became more distinct in both structure and function. These changes occurred not only among sites at depth but also in comparison to shallower communities at the same locations. If only taxonomic β-diversity had increased, it would indicate shifts in species composition without changes in ecological roles (Loiseau et al. 2023). Instead, the combined increases in both taxonomic and functional β-diversity suggest that deeper reef fish communities are undergoing broader ecological shifts in both species and their roles (Loiseau et al. 2023).Due to uneven sampling effort and small sample sizes, it was not possible to robustly identify which species or functional groups shifted in dominance. Nevertheless, at four of the five study sites, deeper fish communities underwent more pronounced ecological shifts. This pattern may reflect environmental changes at 25-40 m, potentially forcing species into shallower habitats, consistent with observed increases in shallow species richness (Medeiros et al. 2021; Smith et al. 2016). Rising sea temperatures have had widespread impacts on Caribbean reefs, with evidence that deeper corals may have lower bleaching thresholds than corals in shallower areas (Smith et al. 2016). Coral bleaching was also documented between our two time periods during the 2016 event in the Bay Islands (Muñiz-Castillo et al. 2024). On deeper reefs elsewhere in the Caribbean, such as Curaçao and Bonaire, declines in calcifying organisms and macroalgae have coincided with increases in cyanobacterial mats and sponge cover (de Bakker et al. 2017). Similar benthic changes may underlie the functional reorganisation observed in deeper reef fish communities, though the absence of benthic data at our sites prevents a direct link. Short-term fluctuations may also play an important role, as in 2020 when hurricanes Eta and Iota struck Honduras and the Bay Islands (Zambrano et al. 2021). Storm damage can have differential impacts on deeper versus shallower reef fish communities (Abesamis et al. 2018). Together, these changes suggest that deeper fish communities, in some cases, are becoming increasingly decoupled from shallower communities, thereby limiting their capacity to serve as refuges (Loiseau et al. 2023; Medeiros et al. 2021; Slattery et al. 2018).

The variability in diversity patterns across sites and depths underscores that several factors, rather than depth alone, play a key role in shaping and maintaining reef fish communities (Kahng et al. 2010). Depth is not an ecological variable in itself but rather a proxy for shifts in other variables such as light availability, temperature, and wave energy (Diaz et al. 2023a,b; Lesser et al. 2009). These, in combination with different types of disturbances, can affect reef communities independently of depth. For instance, shallow areas with greater turbidity or upwelling may experience reduced thermal stress and bleaching during marine heatwaves, which in turn can influence fish dynamics (Randall et al. 2020; Sully and van Woesik 2020; van Woesik et al. 2012). Benthic composition is also a major driver of fish assemblages (Chong-Seng et al. 2012). Therefore, including both benthic data and other abiotic variables in future studies will be important for better understanding temporal changes in fish diversity across depths, as the capacity of deeper reefs to serve as a refuge is likely determined by a combination of these factors (Berryman & Hawkins 2006; Selwood & Zimmer 2020).

The noticeable absence of invasive lionfish in 2022/23, compared to several sightings at 25-40 m in 2014/15, indicates low population sizes around Utila. This is likely due to year-round culling efforts by the numerous dive shops on the island. This trend supports the effectiveness of continuous culling in reducing lionfish density (Barbour et al. 2011; Goodbody-Gringley et al. 2023). However, it is important to note that SVS surveys tend to be biased towards larger, more mobile species and potentially overlook fish species living within the reef structure, such as lionfish, or other smaller species, which means these results may underestimate the abundance and diversity of species inhabiting the reef matrix (Goetze et al. 2019). Advancements in cost-effective remotely operated vehicles (ROVs), bioacoustics, and eDNA could help address the limitations of solely using classic survey methods such as SVS.

This study adds to the evidence questioning the assumption that deeper reefs universally support more stable fish communities and can act as a refuge for their shallow-water counterparts (Bongaerts et al. 2017; Diaz et al. 2023a, b; Loiseau et al. 2023; Medeiros et al. 2021; Rocha et al. 2018; Slattery et al. 2024; Smith et al. 2016). Our findings show that deeper reef communities are still vulnerable to change and often exhibit ecological shifts that do not reflect those seen in adjacent shallow reefs. Because MCEs often harbour distinct species assemblages and functional traits, they cannot be assumed to replenish or stabilise shallow communities following disturbance. Despite their uniqueness, mesophotic communities remain underrepresented in marine conservation strategies (Rocha et al. 2018). Conservation and policy approaches should therefore focus on protecting deeper reefs for their unique biodiversity and ecological functions, rather than solely as refuges for shallower communities (Rocha et al. 2018).

In conclusion, our findings indicate that although α-diversity was less variable at 25-40 m, increases in the β-diversity of dominant species and functional traits (q = 2) suggest that deeper fish communities became increasingly distinct in both structure and function over time. This greater dissimilarity in dominance structure at depth suggests that deeper fish communities are not necessarily less variable. Rather, the extent to which deeper reefs can serve as refuges appears to be highly site-specific and may be influenced by local environmental conditions and stressors as opposed to depth alone.

## Supplementary Information

Below is the link to the electronic supplementary material.

Supplementary file1 (CSV 12 kb)

Supplementary file2 (CSV 8 kb)

Supplementary file3 (DOCX 44 kb)

## Data Availability

All scripts and data needed to replicate the analysis of this study are available on GitHub: https://github.com/James-Boon/Community-change-Coral-Reefs.
