# Supplementary material for: Deeper Caribbean reef fish communities show greater taxonomic and functional change in dominance structure over a nine-year period
Source: Coral Reefs. 2025 Sep 15;45(1):381–94. doi: 10.1007/s00338-025-02709-7 (PMC12916911; doi:10.1007/s00338-025-02709-7)
Supplement: Supplementary file 3 — Supplementary file3 (DOCX 44 kb) [file 338_2025_2709_MOESM3_ESM.docx]

**Deeper Caribbean reef fish communities exhibit increasing taxonomic and functional distinctiveness in dominance structure over a nine-year period - Supplementary**

**Table S1**: Number of 50 m long SVS transects at each site and depth in 2014-2015

|  | **Coral View** | **Little Bight** | **Raggedy Cay** | **Rocky Point** | **The Maze** |
| --- | --- | --- | --- | --- | --- |
| **5 m** | 10 | 10 | 4 | 4 | 10 |
| **15 m** | 9 | 10 | 4 | 4 | 10 |
| **25 m** | 8 | 8 | 4 | 4 | 8 |
| **40 m** | 7 | 7 | 4 | 4 | 8 |

**Table S2**: Number of 50 m long SVS transects at each site and depth in 2022-2023

|  | **Coral View** | **Little Bight** | **Raggedy Cay** | **Rocky Point** | **The Maze** |
| --- | --- | --- | --- | --- | --- |
| **5 m** | 12 | 12 | 12 | 12 | 12 |
| **15 m** | 2 | 12 | 12 | 12 | 12 |
| **25 m** | 8 | 8 | 8 | 8 | 8 |
| **40 m** | 1 | 1 | 7 | 6 | 7 |

**Table S3.** Taxonomic alpha diversity (qTD) of fish communities for diversity orders q = 0 and 2, presented across time periods, depths, and sites. Each estimate includes 95% confidence intervals (qTD.LCL and qTD.UCL) and corresponding sample coverage (SC).

| Site | Depth (m) | Period | Order.q | SC | Method | qTD | qTD.LCL | qTD.UCL |
| --- | --- | --- | --- | --- | --- | --- | --- | --- |
| Coral View | 5_15 | 2014_2015 | 0 | 0.971555 | Rarefaction | 47.56476 | 44.35166 | 50.77787 |
| Coral View | 5_15 | 2014_2015 | 2 | 0.971555 | Rarefaction | 6.13647 | 5.552849 | 6.720091 |
| Coral View | 5_15 | 2022_2023 | 0 | 0.971555 | Rarefaction | 43.98532 | 40.50297 | 47.46768 |
| Coral View | 5_15 | 2022_2023 | 2 | 0.971555 | Rarefaction | 8.10582 | 7.495166 | 8.716473 |
| Little Bight | 5_15 | 2014_2015 | 0 | 0.971555 | Rarefaction | 42.69426 | 39.92063 | 45.46789 |
| Little Bight | 5_15 | 2014_2015 | 2 | 0.971555 | Rarefaction | 11.51211 | 10.75527 | 12.26896 |
| Little Bight | 5_15 | 2022_2023 | 0 | 0.971555 | Rarefaction | 39.80243 | 35.99978 | 43.60508 |
| Little Bight | 5_15 | 2022_2023 | 2 | 0.971555 | Rarefaction | 10.25098 | 9.585708 | 10.91625 |
| Raggedy Cay | 5_15 | 2014_2015 | 0 | 0.971555 | Rarefaction | 24.63048 | 21.55449 | 27.70646 |
| Raggedy Cay | 5_15 | 2014_2015 | 2 | 0.971555 | Rarefaction | 5.302121 | 5.048007 | 5.556236 |
| Raggedy Cay | 5_15 | 2022_2023 | 0 | 0.971555 | Rarefaction | 32.5347 | 30.49251 | 34.57688 |
| Raggedy Cay | 5_15 | 2022_2023 | 2 | 0.971555 | Rarefaction | 4.547364 | 4.328334 | 4.766394 |
| Rocky Point | 5_15 | 2014_2015 | 0 | 0.971555 | Rarefaction | 29.75629 | 27.48025 | 32.03232 |
| Rocky Point | 5_15 | 2014_2015 | 2 | 0.971555 | Rarefaction | 7.670659 | 7.011085 | 8.330234 |
| Rocky Point | 5_15 | 2022_2023 | 0 | 0.971555 | Rarefaction | 38.49193 | 36.44124 | 40.54261 |
| Rocky Point | 5_15 | 2022_2023 | 2 | 0.971555 | Rarefaction | 9.428317 | 8.883918 | 9.972716 |
| The Maze | 5_15 | 2014_2015 | 0 | 0.971555 | Rarefaction | 29.91988 | 27.84896 | 31.99081 |
| The Maze | 5_15 | 2014_2015 | 2 | 0.971555 | Rarefaction | 7.210378 | 6.924388 | 7.496369 |
| The Maze | 5_15 | 2022_2023 | 0 | 0.971555 | Rarefaction | 37.67041 | 35.46143 | 39.87939 |
| The Maze | 5_15 | 2022_2023 | 2 | 0.971555 | Rarefaction | 6.475991 | 6.221996 | 6.729987 |
| Coral View | 25_40 | 2014_2015 | 0 | 0.971555 | Rarefaction | 43.31199 | 38.63562 | 47.98835 |
| Coral View | 25_40 | 2014_2015 | 2 | 0.971555 | Rarefaction | 8.790152 | 7.752426 | 9.827879 |
| Coral View | 25_40 | 2022_2023 | 0 | 0.971555 | Extrapolation | 32.8092 | 21.48889 | 44.12951 |
| Coral View | 25_40 | 2022_2023 | 2 | 0.971555 | Extrapolation | 7.425073 | 5.597469 | 9.252678 |
| Little Bight | 25_40 | 2014_2015 | 0 | 0.971555 | Rarefaction | 47.86525 | 43.72179 | 52.00871 |
| Little Bight | 25_40 | 2014_2015 | 2 | 0.971555 | Rarefaction | 8.519587 | 7.598605 | 9.44057 |
| Little Bight | 25_40 | 2022_2023 | 0 | 0.971555 | Extrapolation | 43.4276 | 23.39089 | 63.46432 |
| Little Bight | 25_40 | 2022_2023 | 2 | 0.971555 | Extrapolation | 14.27176 | 11.89292 | 16.65059 |
| Raggedy Cay | 25_40 | 2014_2015 | 0 | 0.971555 | Rarefaction | 32.1056 | 24.43985 | 39.77136 |
| Raggedy Cay | 25_40 | 2014_2015 | 2 | 0.971555 | Rarefaction | 6.449408 | 5.986613 | 6.912203 |
| Raggedy Cay | 25_40 | 2022_2023 | 0 | 0.971555 | Rarefaction | 40.7247 | 34.46593 | 46.98348 |
| Raggedy Cay | 25_40 | 2022_2023 | 2 | 0.971555 | Rarefaction | 6.423516 | 5.669461 | 7.177572 |
| Rocky Point | 25_40 | 2014_2015 | 0 | 0.971555 | Rarefaction | 43.32027 | 40.60321 | 46.03733 |
| Rocky Point | 25_40 | 2014_2015 | 2 | 0.971555 | Rarefaction | 4.691547 | 4.2366 | 5.146494 |
| Rocky Point | 25_40 | 2022_2023 | 0 | 0.971555 | Rarefaction | 47.6598 | 35.89378 | 59.42582 |
| Rocky Point | 25_40 | 2022_2023 | 2 | 0.971555 | Rarefaction | 5.322752 | 4.442911 | 6.202592 |
| The Maze | 25_40 | 2014_2015 | 0 | 0.971555 | Rarefaction | 36.17246 | 31.41566 | 40.92927 |
| The Maze | 25_40 | 2014_2015 | 2 | 0.971555 | Rarefaction | 5.747002 | 5.25517 | 6.238834 |
| The Maze | 25_40 | 2022_2023 | 0 | 0.971555 | Rarefaction | 36.74644 | 30.89271 | 42.60016 |
| The Maze | 25_40 | 2022_2023 | 2 | 0.971555 | Rarefaction | 8.003801 | 6.93455 | 9.073053 |

**Table S4.** Functional alpha diversity (qFD) of fish communities for diversity orders q = 0 and 2, presented across time periods, depths, and sites. Each estimate includes 95% confidence intervals (qFD.LCL and qFD.UCL) and corresponding sample coverage (SC).

| Site | Depth | Period | Order.q | SC | Method | qFD | qFD.LCL | qFD.UCL |
| --- | --- | --- | --- | --- | --- | --- | --- | --- |
| Coral View | 25_40 | 2014_2015 | 0 | 0.9715554 | Rarefaction | 10.08043499 | 9.135469315 | 11.02540067 |
| Coral View | 25_40 | 2022_2023 | 0 | 0.9715554 | Extrapolation | 7.392879235 | 6.286779762 | 8.498978707 |
| Coral View | 5_15 | 2014_2015 | 0 | 0.9715554 | Rarefaction | 9.660795526 | 9.309891783 | 10.01169927 |
| Coral View | 5_15 | 2022_2023 | 0 | 0.9715554 | Rarefaction | 9.177856294 | 8.601094667 | 9.75461792 |
| Little Bight | 25_40 | 2014_2015 | 0 | 0.9715554 | Rarefaction | 10.15558979 | 9.405900337 | 10.90527924 |
| Little Bight | 25_40 | 2022_2023 | 0 | 0.9715554 | Extrapolation | 9.312167135 | 1.836344384 | 16.78798989 |
| Little Bight | 5_15 | 2014_2015 | 0 | 0.9715554 | Rarefaction | 8.796914532 | 8.53667159 | 9.057157473 |
| Little Bight | 5_15 | 2022_2023 | 0 | 0.9715554 | Rarefaction | 8.808120781 | 8.187311889 | 9.428929672 |
| Raggedy Cay | 25_40 | 2014_2015 | 0 | 0.9715554 | Rarefaction | 7.778675692 | 5.287869027 | 10.26948236 |
| Raggedy Cay | 25_40 | 2022_2023 | 0 | 0.9715554 | Rarefaction | 8.587519418 | 8.023180991 | 9.151857845 |
| Raggedy Cay | 5_15 | 2014_2015 | 0 | 0.9715554 | Rarefaction | 7.550413024 | 6.909455375 | 8.191370673 |
| Raggedy Cay | 5_15 | 2022_2023 | 0 | 0.9715554 | Rarefaction | 7.985103243 | 7.660582703 | 8.309623783 |
| Rocky Point | 25_40 | 2014_2015 | 0 | 0.9715554 | Rarefaction | 8.440547291 | 8.033389151 | 8.847705431 |
| Rocky Point | 25_40 | 2022_2023 | 0 | 0.9715554 | Rarefaction | 8.537350337 | 6.816833575 | 10.2578671 |
| Rocky Point | 5_15 | 2014_2015 | 0 | 0.9715554 | Rarefaction | 7.921466573 | 7.660494753 | 8.182438392 |
| Rocky Point | 5_15 | 2022_2023 | 0 | 0.9715554 | Rarefaction | 8.282744894 | 7.732720372 | 8.832769415 |
| The Maze | 25_40 | 2014_2015 | 0 | 0.9715554 | Rarefaction | 7.826128281 | 6.33359369 | 9.318662872 |
| The Maze | 25_40 | 2022_2023 | 0 | 0.9715554 | Rarefaction | 8.280594673 | 7.420962292 | 9.140227054 |
| The Maze | 5_15 | 2014_2015 | 0 | 0.9715554 | Rarefaction | 7.819973231 | 7.4348449 | 8.205101562 |
| The Maze | 5_15 | 2022_2023 | 0 | 0.9715554 | Rarefaction | 8.820195954 | 8.512650613 | 9.127741294 |
| Coral View | 25_40 | 2014_2015 | 2 | 1 | Extrapolation | 4.545854723 | 4.256774841 | 4.834934606 |
| Coral View | 25_40 | 2022_2023 | 2 | 1 | Extrapolation | 3.656391647 | 3.136228438 | 4.176554855 |
| Coral View | 5_15 | 2014_2015 | 2 | 1 | Extrapolation | 3.371389372 | 3.151981465 | 3.59079728 |
| Coral View | 5_15 | 2022_2023 | 2 | 1 | Extrapolation | 3.963734814 | 3.780786912 | 4.146682716 |
| Little Bight | 25_40 | 2014_2015 | 2 | 1 | Extrapolation | 4.237932106 | 3.968855247 | 4.507008965 |
| Little Bight | 25_40 | 2022_2023 | 2 | 1 | Extrapolation | 5.498679592 | 4.901526157 | 6.095833027 |
| Little Bight | 5_15 | 2014_2015 | 2 | 1 | Extrapolation | 5.045128617 | 4.863043274 | 5.227213961 |
| Little Bight | 5_15 | 2022_2023 | 2 | 1 | Extrapolation | 4.464336729 | 4.263593643 | 4.665079816 |
| Raggedy Cay | 25_40 | 2014_2015 | 2 | 1 | Extrapolation | 3.193174741 | 3.036752046 | 3.349597437 |
| Raggedy Cay | 25_40 | 2022_2023 | 2 | 1 | Extrapolation | 2.609727833 | 2.376083247 | 2.843372419 |
| Raggedy Cay | 5_15 | 2014_2015 | 2 | 1 | Extrapolation | 3.235040747 | 3.101978552 | 3.368102941 |
| Raggedy Cay | 5_15 | 2022_2023 | 2 | 1 | Extrapolation | 2.748234876 | 2.661734827 | 2.834734926 |
| Rocky Point | 25_40 | 2014_2015 | 2 | 1 | Extrapolation | 2.937393308 | 2.795018608 | 3.079768009 |
| Rocky Point | 25_40 | 2022_2023 | 2 | 1 | Extrapolation | 2.893088512 | 2.619082597 | 3.167094427 |
| Rocky Point | 5_15 | 2014_2015 | 2 | 1 | Extrapolation | 3.99971112 | 3.788483714 | 4.210938525 |
| Rocky Point | 5_15 | 2022_2023 | 2 | 1 | Extrapolation | 4.436648594 | 4.316680775 | 4.556616412 |
| The Maze | 25_40 | 2014_2015 | 2 | 1 | Extrapolation | 2.358783425 | 2.185631998 | 2.531934852 |
| The Maze | 25_40 | 2022_2023 | 2 | 1 | Extrapolation | 2.927959058 | 2.654733739 | 3.201184377 |
| The Maze | 5_15 | 2014_2015 | 2 | 1 | Extrapolation | 3.853390054 | 3.732592552 | 3.974187555 |
| The Maze | 5_15 | 2022_2023 | 2 | 1 | Extrapolation | 3.513954887 | 3.428992714 | 3.59891706 |

| Site | Depth (m) | Beta q=0 | Beta LCL q=0 | Beta UCL q=0 | Beta q=2 | Beta LCL q=2 | Beta UCL q=2 |
| --- | --- | --- | --- | --- | --- | --- | --- |
| Coral View | 5_15 | 1.2256 | 1.0988 | 1.3524 | 1.301 | 1.2667 | 1.3353 |
| Little Bight | 5_15 | 1.2368 | 1.1314 | 1.3422 | 1.0798 | 1.0589 | 1.1007 |
| Raggedy Cay | 5_15 | 1.1785 | 1.0277 | 1.3292 | 1.251 | 1.236 | 1.266 |
| Rocky Point | 5_15 | 1.2943 | 1.0448 | 1.5437 | 1.0604 | 1.0496 | 1.0713 |
| The Maze | 5_15 | 1.1532 | 1.0746 | 1.2318 | 1.0266 | 1.0204 | 1.0328 |
| Coral View | 25_40 | 1.7147 | 1.596 | 1.8333 | 1.4276 | 1.3623 | 1.493 |
| Little Bight | 25_40 | 1.2586 | 1.0396 | 1.4777 | 1.6217 | 1.5792 | 1.6642 |
| Raggedy Cay | 25_40 | 1.5469 | 1.3703 | 1.7235 | 1.156 | 1.1254 | 1.1865 |
| Rocky Point | 25_40 | 1.2588 | 0.9416 | 1.576 | 1.6275 | 1.5767 | 1.6783 |
| The Maze | 25_40 | 1.1516 | 0.8136 | 1.4896 | 1.0837 | 1.0625 | 1.1048 |

**Table S5.** Taxonomic beta diversity (q = 0 and 2) of fish communities across depths and sites. Estimates reflect changes between fish communities between two time periods (2014/15 and 2022/23) within the same depth and site. Values include 95% confidence intervals (lower and upper: LCL and UCL).

**Table S6.** Functional beta diversity (q = 0 and 2) of fish communities across depths and sites. Estimates reflect changes between fish communities between two time periods (2014/15 and 2022/23) within the same depth and site. Values include 95% confidence intervals (lower and upper: LCL and UCL).

| Site | Depth (m) | Beta q=0 | Beta LCL q=0 | Beta UCL q=0 | Beta q=2 | Beta LCL q=2 | Beta UCL q=2 |
| --- | --- | --- | --- | --- | --- | --- | --- |
| Coral View | 5_15 | 0.9996 | 0.9983 | 1.0008 | 1.1755 | 1.1453 | 1.2058 |
| Little Bight | 5_15 | 1 | 0.9939 | 1.0061 | 1.0325 | 1.0231 | 1.0419 |
| Raggedy Cay | 5_15 | 1.0074 | 0.9996 | 1.0153 | 1.2003 | 1.1843 | 1.2162 |
| Rocky Point | 5_15 | 0.9973 | 0.9862 | 1.0084 | 1.0418 | 1.0373 | 1.0462 |
| The Maze | 5_15 | 1.0021 | 0.9981 | 1.006 | 1.0067 | 1.0041 | 1.0094 |
| Coral View | 25_40 | 1.008 | 0.9967 | 1.0193 | 1.3408 | 1.3138 | 1.3679 |
| Little Bight | 25_40 | 0.9878 | 0.9587 | 1.0169 | 1.5004 | 1.4752 | 1.5256 |
| Raggedy Cay | 25_40 | 1.0089 | 0.9469 | 1.0709 | 1.0556 | 1.0441 | 1.0671 |
| Rocky Point | 25_40 | 1.0004 | 0.9898 | 1.011 | 1.463 | 1.4373 | 1.4887 |
| The Maze | 25_40 | 1.0141 | 0.9905 | 1.0378 | 1.0533 | 1.0354 | 1.0713 |
